# Obstructive sleep apnea risk and its associated factors among type 2 diabetes mellitus patients at wolkite university specialized hospital, Wolkite, Southern Ethiopia, 2021. A comparative cross-sectional study

**DOI:** 10.1186/s13098-022-00931-9

**Published:** 2022-10-27

**Authors:** Alemayehu Wondie, Mitku Mammo Taderegew, Betemariam Girma, Atsede Getawey, Daniel Tsega, Tamene Fetene Terefe, Shimelis Mitiku, Hiwot Berhanu

**Affiliations:** 1 Department of Biomedical Sciences, College of Medicine and Health Science, Wolkite University, P.O. Box 07, Wolkite, Ethiopia; 2 Department of Midwifery, College of Medicine and Health Sciences, Wolkite University, Wolkite, Ethiopia; 3 Department of Nursing, College of Medicine and Health Sciences, Wolkite University, Wolkite, Ethiopia; 4 Department of Biomedical Sciences, Institute of Health, Jimma University, Jimma, Ethiopia

**Keywords:** Obstructive sleep apnea, Type 2 Diabetes Mellitus, Berlin questionnaire, Neck grasp

## Abstract

**Background:**

Obstructive sleep apnea is a syndrome characterized by recurrent partial, or complete upper airway collapse during sleep. Although obstructive sleep apnea is common in type 2 diabetes mellitus, the majority of patients remain undiagnosed because of the prohibitive cost of the test and paucity of the sleep clinic, especially in developing nations. The study aimed to assess high-risk obstructive sleep apnea and its associated factors among type 2 diabetes mellitus patients at Wolkite University Specialized Hospital.

**Methods:**

A Hospital**-**based comparative cross-sectional study was employed from October 15 to December 5, 2021, among 204 participants. Data collection was done by semi-structured interviewer-administered questionnaires. Data was entered into the Epi data version 4.6 and exported to SPSS version 25.0 for analysis. Independent t-test and chi-square test were used to compare continuous and categorical variables accordingly. Binary and multiple logistic regression analysis was used to assess factors associated with high-risk obstructive sleep apnea. Statistical significance was set at P-value < 0.05.

**Results:**

A total of 204 participants with an equal proportion of the two groups were included with a 100% response rate. About 56.9% of the participants were males. The mean age of type 2 diabetes mellitus patients was 57.1 (± 12.0) years and the non-diabetic group was 55.3 (± 10.9) years. The prevalence of high-risk obstructive sleep apnea among type 2 diabetes mellitus was 42.2%, and that of non-diabetics was 13.7% (p < 0.001). Age (AOR: 1.13; 95%CI: 1.04, 1.22), neck grasp (AOR: 6.48; 95%CI: 1.56, 26.96), waist circumference (AOR: 4.44; 95%CI: 1.12, 17.61) and the presence of diabetes-related complications (AOR: 4.18; 95%CI: 1.13, 15.43) were independently associated with high-risk obstructive sleep apnea among type 2 diabetes mellitus patients.

**Conclusion:**

The prevalence of high-risk obstructive sleep apnea among type 2 diabetes mellitus was higher with a significant difference from their comparison group. Age, neck grasp, waist circumference, and diabetes-related complications were significantly associated with high-risk obstructive sleep apnea among type 2 diabetes mellitus patients. Therefore, type 2 diabetes mellitus patients should be screened for obstructive sleep apnea in the early course of their follow-up to take preventive measures and early treatments.

## Background

Sleep is a complex biological state which is essential in life and characterized by changes in behavioral, physiological, and electrophysiological parameters. A failure in sleep regulation in the brain is associated with distinct physiological alterations that influence upper airway function; a decrease in adrenergic and serotonergic control of the pharyngeal dilator muscles with accompanying cholinergic-mediated suppression of genioglossus activity leads to sleep disorder breathing [1–4]. Sleep disorder breathing (SDB) events are classified further into obstructive, central, and mixed events based on the cause and respiratory effort that persists in the absence of airflow. Obstructive sleep apnea (OSA) is a syndrome characterized by recurrent partial, or complete upper airway collapse during sleep which leads to intermittent hypoxia and sleep fragmentation. It is the most common form of SDB accounting for about 85% of the cases [5, 6].

Diabetes Mellitus (DM) affects an estimated 10.5% of adults worldwide and about 6.7 million deaths are attributed to DM annually from which type 2 diabetes mellitus (T2DM) constitutes up to 90% of all the cases [7]. T2DM can predispose to OSA, in turn, OSA can predispose to insulin resistance and T2DM. Intermittent hypoxemia and sleep fragmentation, the two cardinal features of OSA lead to derangement in glucose metabolism by the alteration of the neuroendocrine system. Sympathetic nervous system activation, hypothalamic-pituitary axis alterations, adipokine disturbances, systemic inflammation, and oxidative stress results in insulin resistance and T2DM. On the other hand, peripheral neuropathy, insulin resistance, leptin resistance, and overall oxidative stress, which are consequences of diabetes mellitus, may alter the neuronal and mechanical control of the upper airway muscles, leading to easy collapsibility of these muscles during sleep that causes OSA [8–10].

Clinical symptoms suggestive of OSA can be divided into nocturnal symptoms and daytime symptoms. The nocturnal symptoms include frequent snoring, breathing pauses, choking/gasping, insomnia and paroxysmal nocturnal dyspnea. The daytime symptoms include morning headache, excessive daytime sleepiness, irritability, memory lapses and decreased concentration [11].

The definitive diagnosis of OSA is done with polysomnography (PSG), based on the apnea-hypopnea index (AHI). An AHI, which is the average number of apnea and hypopnea episodes per hour during sleep, ≥ 5 events/hour is diagnosed as OSA consistent with typical symptoms of OSA [6]. However, polysomnography is time-consuming, expensive, and not easily available everywhere. The prohibitive cost of the test and paucity of the sleep laboratory in developing nations limit its access. So, utilizing another simple and economical screening tool for the identification of OSA at-risk patients is of special public health concern to reduce the OSA-related burdens. Several clinical predictors and questionnaires are also used to screen for or diagnose OSA, some of which include Berlin questionnaire, Epworth sleepiness scale, and STOP-BANG questionnaire [12].

The Berlin Questionnaire has been used expansively in clinical and epidemiologic research internationally to screen OSA risk and there is high consistency between results of high-risk OSA by the Berlin Questionnaire, and OSA by the parameters of PSG for the early identification of OSA among T2DM patients [12–15].

Obstructive sleep apnea and T2DM are common and worsening global health problems [16]. OSA is estimated to affect 936 million adults aged 30–69 years worldwide. It is currently recognized as an important health issue that can affect a variety of organs in the cardiovascular, neurologic, respiratory, and endocrine systems [17, 18].

Obstructive sleep apnea is a common, under-recognized, under-diagnosed, under-treated, and serious medical condition in adults, affecting millions of adults worldwide. Unfortunately, up to 90% of individuals with OSA remain without diagnosis or therapy [19, 20]. Study shows high prevalence of obstructive sleep apnea in patients with insulin resistance conditions than in the general population which was estimated in the range of 9–32.8%. Persons with T2DM are at increased risk of obstructive sleep apnea with a reported prevalence ranging from 23–90.7% [21–26].

The existence of a link between OSA and T2DM share common risk factors such as obesity and age, which are also risk factors for cardiovascular diseases; such as heart failure, coronary artery disease, arrhythmia and sudden death [27]. Individuals with comorbid T2DM and OSA have higher blood pressure, poor sleep quality, lower health-related quality of life, and lower adherence to diabetes self-management practices [28]. The study displayed that about 55.5% of OSA among T2DM patients had at least one diabetes-related complication which was mediated by the presence of hypertension, as post hoc power of the test estimated, 73%, 93.3%, and 52.1% for OSA leads to hypertension, hypertension mediates T2DM-related complications, and OSA leads directly to diabetes-related complications, respectively [29].

Even though Screening for OSA in patients with diabetes is recommended by the American Diabetes Association, the frequency which is done in clinical practice is unknown. Large discrepancies between expected and diagnosed cases of OSA in patients with diabetes have been reported, suggesting that the majority of patients at risk for OSA are not being identified [30, 31].

Up to the authors' search catalog, there is no comparative study is done in Ethiopia to determine the significant difference in high-risk OSA between T2DM patients, and the non-diabetic population. In addition, the prevalence of high-risk OSA and its association among diabetic societies in southern Ethiopia is not studied yet. OSA screening practices in T2DM patients on follow-up at Wolkite University specialized hospital (WUSH) have not been previously explored. Therefore, this study aimed to assess high-risk OSA among T2DM patients and their comparison group, and factors associated with high-risk OSA among T2DM patients on follow-up at WUSH.

This study will create an awareness of the OSA-related burden among T2DM societies to take preventive measures and publicize physicians to use OSA risk screening tools in clinical practice for diagnosis, treatment and to prevent OSA-related complications. This finding will be used as baseline data for the hospital administrative office for the implementation of OSA screening tools in clinical practice to improve OSA-related disorders. It can also provide additional information for the scientific community for further study and recommendations.

## Methods

### Study setting and design

A comparative cross-sectional study was conducted in WUSH, which is located in Gubre sub-city in wolkite town, Gurage, Southern Ethiopia. It is found at a distance of 172 km from Addis Ababa, the capital city of Ethiopia 14 km along the road of Butta Jira from wolkite town. The hospital is established in 2018 as a part of a teaching hospital for health science students to produce qualified health professionals by providing practical skills. The hospital delivers health services for about 4 million catchment population living in Gurage zone and Yem special woreda both inpatient as well as an outpatient department (OPD). The hospital has two DM follow-up OPD rooms for diabetic patients to take regular follow-up, counseling, and treatment during all working hours of the day. At the time of the data collection, about 470 T2DM patients had regular follow-ups. The study was conducted from October 15 to December 5, 2021.

### Study population and sampling techniques

All T2DM patients age greater than 18 on follow-up who visits the hospital were selected through consecutive sampling technique for the study group, Purposive sampling technique was used for age-sex matched non-diabetic participants for the comparison group who came to the hospital during the study period were included until the required sample size was attained.

Study participants which didn’t fulfill inclusion criteria; T2DM patients who were pregnant, any critical illness, patients who had follow-up for the second time during data collection, bronchial asthma, COPD, bronchitis, lung cancer and patients with neck mass were excluded.

The sample size was determined by using STAT CALC Epi-info version 7.0.8.3 for two population cross-sectional study to get the maximum sample size, considering a two-sided confidence level of 95%, power of 80%, and a 1:1 ratio of T2DM patients and non-diabetic comparison group. The prevalence of high-risk OSA in T2DM patients (79%) and non-diabetic participants (60%), was taken from a comparative cross-sectional study done in India [32]. The total sample size was 204 participants (102 T2DM and 102 non-diabetics).

### Data collection procedures

Three skilled personnel (two BSc nurses and one laboratory technologist) were selected and adequate training was delivered by the principal investigator (PI) for 2 days about the aim of the study, data collection tools, types, and procedures of anthropometric measurements that were included. The PI also delivered two questionnaires each for data collectors to self-exercise before the actual data collection was started. Questionnaires were initially prepared in English and then translated to the Amharic language then translated to English to ensure its consistency.

A pretest was done on 5% of the sample population of a similar setting at Wolkite Health Center which was not included in the study population before conducting the study and necessary corrections were taken concerning wording and contextual variations on the semi-structured questionnaires. Again, to assure the quality of the data interview were also done with respondent family members as a witness and all the respondents included were aged more than 18. The data collection procedures and completeness of the interviewed questionnaires were checked frequently through supervision for their consistency on each day by the investigator.

Data were collected by berlin questionnaires adopted from German and translated into Amharic. The Berlin questionnaire was an outcome of the Conference on Sleep in Primary Care, held in April 1996 in Berlin, Germany [33]. Berlin Questionnaire includes 10 items of questions classified into three categories which aimed to detect important signs and symptoms for the diagnosis of high-risk OSA. Category 1 includes five questions on snoring and witnessed apnea. Snoring-related questionnaires include the presence of snoring, frequency of snoring, and type of snoring were considered to have frequent snoring if the Participant had snoring that occurred at least three to four times per week regardless of the snoring type. Category 1, was considered positive if two or more questions are reported positive at least three to four times/week. Category 2 includes three questions that assess participants’ tiredness and drowsiness. Category 2 was considered positive if at least two of the three questions were reported positive at least three to four times/week. Category 3 comprises the blood pressure status and BMI of the participants. Category 3 was positive if participants have been diagnosed as hypertensive or had BMI greater or equal to 30 kg/m^2^. Participants were instructed to answer questions based on the interviewer-administered Berlin questionnaire accordingly. If the participants scored positive in at least two of the three categories were considered to have high-risk OSA. If the participant scored positive in only one or none of the three categories, were determined to have low-risk OSA [34, 35].

Blood pressure was measured by a digital sphygmomanometer (DX-81, China) three times after at least five minutes of rest in the sitting position from the left arm while placing the hand at the level of the heart and the average was taken to classify hypertension. Blood pressure ≥ 140/90 or known hypertensive patient who is on medication regardless of the current BP were considered to have hypertension.

Height was measured to the nearest 0.5 cm using a fixed stadiometer with a vertical backboard and a moveable headboard. Weight was taken to the nearest 0.5 kg with a digital weight scale after rough calibration to ensure it was functioning properly in which the participant stands in a normal anatomic position. Body Mass Index (BMI) computed from height and weight by SPSS software was used as a screening tool to measure the magnitude of obesity among participants. Based on WHO BMI classification criteria, BMI greater or equal to 30, 25–29.99 were considered obese and overweight respectively [13].

Waist circumference was measured to determine central obesity. The measuring tape was put at the level midway between the iliac crest and the lower rib margin on a horizontal scale. The Waist circumference of > 88 cm for women and > 102 cm for men were considered central obesity [36].

Neck circumference was measured to the nearest 0.5 cm using measuring tape around the neck at the middle of the neck between the mid-cervical spine and the mid-anterior neck 0.5 cm below the most prominent portion of the thyroid cartilage, the laryngeal prominence. Neck circumference of 43 cm for men and 40 cm for women were a cut point for high Neck circumference [26].

A neck grasp gap measure was taken to determine its independent association with OSA. Neck grasp was measured by assisting the patients to encircle their hands around the neck. Neck grasp entails placing both thumbs together at the anterior of the neck and encircling the fingers in the posterior. Positive neck grasp, if the patient is unable to encircle their neck resulting in at least a 1 cm gap between fingertips, and Negative neck grasp if the patient can encircle their neck defined as touching fingertips in the posterior neck [26].

A drop of blood sample was taken from the finger prick in an aseptic condition and FBS was measured by the digital glucometer (GM505PAD, Korea) after overnight fasting. For T2DM patients, the average of three consecutive follow-up measurements (two from prior record and one from current FBS record) was used to determine blood glucose control level. Blood glucose ≥ 126 mg/dl was considered poorly controlled. FBS for the comparison group was done to confirm them as non-diabetic (FBS < 100 mg/dl).

### Operational definitions

Central obesity: a person whose waist circumference of > 88 cm for women and > 102 cm for men. Critically ill: Patients who are unable to communicate and have abnormal consciousness.

Frequent Snoring: a coarse and vibratory sound during sleep that occurred at least 3–4 times/week.

High-risk OSA: a syndrome diagnosed with a positive berlin score in at least two of the three categories.

Low-risk OSA: Person who had berlin score positive in only one or none of the three categories.

Non-diabetes: a person whose fasting blood glucose level is less than 100 mg/dl.

Behavioral factors: the taking of substances that can quit or change theirs to unhealthy.

Diabetes-related complication: patients who had at least one known microvascular complication in their follow-up record.

### Data processing and analysis

The collected data were cleaned for completeness and consistency, edited, and compiled. Response in each question was coded for simplicity of data entry. The data entry was carried out by using the Epi data version 4.6 and exported to SPSS version 25.0 for analysis. Exported data were checked for missing values and outliers. Independent sample t-test and chi-square test were used to compare continuous and categorical variables accordingly. Data were described by the text, frequency, tables, and graphs.

Binary logistic regression analysis was used to determine the crude relationship between high-risk OSA and some independent variables that were expressed in the confidence interval, odds ratio, and p-value. Variables with a p-value of less than 0.25 were candidates for multiple logistic regression analysis models by using the backward stepwise method to examine the net effect of each independent variable on a dependent variable expressed in an adjusted odds ratio, confidence interval, and p-value. Model fitness was verified by Hosmer and Lemeshow test (p-value = 0.554) and multicollinearity of the variables was checked via tolerance test and variance inflation factor. Statistical significance was set at P-value < 0.05.

## Results

### Socio-demographic characteristics of the participants

In this study, 204 (102 T2DM and 102 non-diabetic) participants matched in age and sex with a 100% response rate were recruited. About 58 (56.9%) participants in each group were males. The mean and standard deviation of the age of T2DM patients was 57.1 (± 12.0) years, and their comparison group was 55.3 (± 10.9) years. Regards to the level of education, about 20 (19.6%) of T2DM patients, and 22 (21.6%) of the non-diabetic participants were illiterates while 40 (39.2%) of each group attend secondary education and above. The majority of participants, 91 (89.2%) of T2DM and 83 (81.4%) non-diabetic participants were urban residents (Table 1).

**Table 1:** Sociodemographic characteristics of the respondents in WUSH, Southern Ethiopia, 2021

Variables	Category	T2DM (n = 102) (n, %)	Non-diabetic (n = 102) (n, %)	p-value
Sex	Male	58 (56.9)	58 (56.9)	1.000
	Female	44 (43.1)	44 (43.1)	
Age (years), mean (± SD)		57.1 (12.0)	55.3 (10.9)	0.263
Marital status	Married	80 (78.4)	87 (85.3)	0.478
	Divorced	3 (2.9)	1 (1)	
	Widowed	13 (12.7)	8 (7.8)	
	Single	6 (5.9)	6 (5.9)	
Occupation	Farmer	10 (9.8)	11 (10.8)	0.124
	G/ E	19 (18.6)	30 (29.4)	
	Merchant	48 (47.1)	37 (36.3)	
	Housewife	19 (18.6)	11 (10.8)	
	Others*	6 (5.9)	13 (12.7)	
Educational status	Illiterate	20 (19.6)	22 (21.6)	0.931
	Primary	42 (41.2)	40 (39.2)	
	Secondary & above	40 (39.2)	40 (39.2)	
Residence	Urban	91 (89.2)	83 (81.4)	0.114
	Rural	11 (10.8)	19 (18.6)	

*G/E* Government Employee, *SD* Standard Deviation

^*^Daily labor & non-employed

### Anthropometry, clinical parameters & behavioral factors

The mean BMI (± SD) of the study group was 26.1 (± 4.2) kg/m^2^, and their comparison was 26.5 (± 3.3) kg/m^2^. About 45 (44.1%) of the study group and 42 (41.2%) of its comparison had central obesity. As of the neck gap measure, 35 (34.3%) of T2DM patients and 13 (12.7%) of the comparison group had positive neck grasp.

Appraise of behavioral factors, 73 (71.6%) of T2DM patients, and 64 (62.7%) of their comparison use at least one substance either currently or formerly in their lifetime. Of those 31 (30.45) of the study group, and 29 (28.4%) of their comparison were current alcohol drinkers. In addition, only 8 (7.8%) of T2DM patients, and 9(8.8%) of non-diabetes had a history of smoking (Table 2).

**Table 2:** Anthropometry, and behavioral factors of participants in WUSH, southern Ethiopia, 2021

Variables	Category	T2DM (n, %)	Non-diabetic (n, %)	p-value
Waist circumference (cm)	Central obesity	45 (44.1)	42 (41.2)	0.671
	Normal	57 (55.9)	60 (58.9)	
Neck Circumference (cm)	High	34 (33.3)	21 (20.6)	0.040
	Normal	68 (66.7)	81 (79.4)	
Neck grasp	Positive	35 (34.3)	13 (12.7)	< 0.001
	Negative	67 (65.7)	89 (87.3)	
Body Mass Index	Obesity	26 (25.5)	14 (13.7)	0.042
	Overweight	35 (34.3)	50 (49)	
	Normal	41 (40.2)	38 (37.3)	
Blood glucose level (mg/dl), mean (± SD)		171.1 (62.5)	87.1 (17.2)	< 0.001
Alcoholic beverages	Former drinker	16 (15.7)	10 (9.8)	0.369
	Current	31 (30.4)	29 (28.4)	
	Never	55 (53.9)	63 (61.8)	
khat chewers	Former	10 (9.8)	4 (3.9)	0.189
	Current	24 (23.5)	21 (20.6)	
	Never	68 (66.7)	77 (75.5)	
Smoking	Former	7 (6.9)	2 (2.0)	0.026
	Current	1 (1.0)	7 (6.9)	
	Never	94 (92.2)	93 (91.2)	
Sleeping pills/antipain user	Former	20 (19.6)	10 (9.8)	0.074
	Current	20 (19.6)	16 (15.7)	
	Never	62 (60.8)	76 (74.5)	

T2DM; Type 2 Diabetes Mellitus, SD; standard deviation

Regards to T2DM-related parameters, about 49 (48%) of the study group had a history of T2DM for more than five years. The majority, 88 (86.3%) of T2DM patients used oral hypoglycemic agents alone and 13 (12.7%) used a combination therapy of insulin and oral hypoglycemic agent. About 44(43.1%) of T2DM patients had known comorbidity, of those majority, 39(88.6%) was comorbid hypertension and 6 (13.6%) was kidney disease. More than a quarter 29 (28.4%) of the study group had known T2DM-related complications, of that 19 (65.5%) was diabetic neuropathy (Table 3).

**Table 3:** T2DM-related variables among T2DM patients in WUSH, southern Ethiopia, 2021

T2DM-related Variables	Category	Frequency (n)	Percent (%)
Duration (years)	> 5	49	48
	≤ 5	53	52
Blood glucose control level	Poor control	78	76.5
	Good control	24	23.5
Comorbidity	Yes	44	43.1
	No	58	56.9
Medications	Oral alone	88	86.3
	Oral and insulin	13	12.7
	Non-pharmacologic	1	1.0
T2DM-related complications	Yes	29	28.4
	No	73	71.6
Types of complications	Diabetic neuropathy	19	65.5
	Diabetic retinopathy	8	27.6
	Diabetic nephropathy	2	6.9

*T2DM* Type 2 Diabetes Mellitus

### Prevalence of obstructive sleep apnea risk among participants

**Fig. 1:**
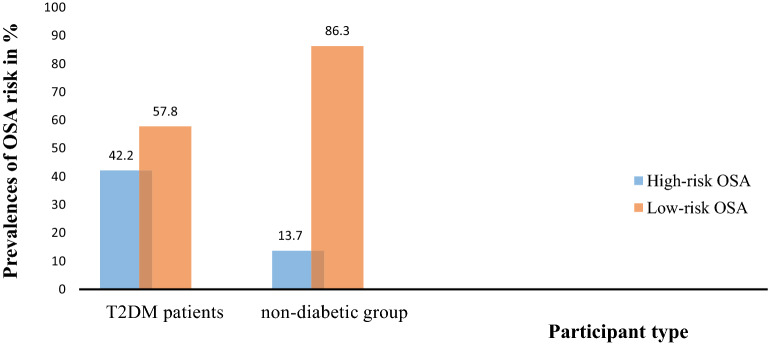
Graphic presentation of OSA risk among T2DM patients and non-diabetic participants

The prevalence of High-risk OSA among T2DM patients was 42.2% (95% CI: 32.4, 51.9) while 57.8% of T2DM patients had low-risk OSA. The prevalence of high-risk OSA among non-diabetic participants was 13.7% (95% CI: 6.9, 20.5) and the rest 86.3% had low-risk OSA (Fig. 1).

According to berlin score to diagnose OSA risk based on the parameter of signs and symptoms of OSA, 48 (47.1%), and 7 (6.8%) T2DM patients reported frequent snoring and breathing pauses during sleep, respectively as compared to 33 (32.4%) frequent snoring and 1 (1%) breathing pause among non-diabetic participants. Of T2DM patients who had frequent snoring, 29 (60.4%) was snoring slightly louder than breathing, and 17 (35.4%) was snoring as loud as talking as compared to the majority, 30 (90%) snoring was slightly louder than breathing among non-diabetics. Category one positive result was scored on 37 (36.3%) T2DM patients, and 17 (16.7%) non-diabetic participants. Positive category two result was scored among 40 (39.2%) T2DM patients and 7 (6.9%) non-diabetic participants from the parameters of tiredness and drowsiness. For the category three result, about 39 (38.2%) of T2DM patients and 22 (21.6%) of the non-diabetic group had hypertension. Obesity was recorded in 26 (25.5%) T2DM patients, and 14 (13.7%) non-diabetic group. From the two parameters, 49 (48%) T2DM patients and 32 (31.4%) non-diabetic participants scored positive for category three (Table 4).

**Table 4:** Berlin score of OSA risk among respondents in WUSH, southern Ethiopia, 2021

Variables		T2DM, n = 102 (n, %)	Non-diabetics, n = 102 (n, %)	P-value
Frequent snoring*	Yes	48 (47.1)	33 (32.4)	0.032
	No	54 (52.9)	69 (67.6)	
Snoring bothered others*	Yes	36 (35.3)	18 (17.6)	0.004
	No	66 (64.7)	84 (82.4)	
Breathing pause during sleep*	Yes	7 (6.8)	1 (1.0)	0.030
	No	95 (93.2)	101 (99.0)	
Category 1 result	Positive	37 (36.3)	17 (16.7)	0.002
	Negative	65 (63.7)	85 (83.3)	
Feel tired after sleep**	Yes	44 (43.1)	15 (14.7)	< 0.001
	No	58 (56.9)	87 (85.3)	
Feel tired during wake time**	Yes	40 (39.2)	11 (10.8)	< 0.001
	No	62 (60.8)	91 (89.2)	
Frequent nodding-off while in car**	Yes	16 (15.7)	7 (6.9)	0.046
	No	86 (84.3)	95 (93.1)	
Category 2 result	Positive	40 (39.2)	7 (6.9)	< 0.001
	Negative	62 (60.8)	95 (93.1)	
Hypertension***	Yes	39 (38.2)	22 (21.6)	0.009
	No	63 (61.8)	80 (78.4)	
Obesity***	Yes	26 (25.5)	14 (13.7)	0.034
	No	76 (75.5)	88 (86.3)	
Category 3 result	Positive	49 (48)	32 (31.4)	0.015
	Negative	53 (52)	70 (68.6)	
OSA risk	High-risk	43 (42.2)	14 (13.7)	< 0.001
	Low-risk	59 (57.8)	88 (86.3)	

*OSA* Obstructive Sleep Apnea, *T2DM* Type 2 Diabetes Mellitus

Category 1 result computed from the three variables labeled in *, Category 2 result computed from the three variables labeled in ** and Category 3 result computed from the last two variables labeled in***

**Fig. 2:**
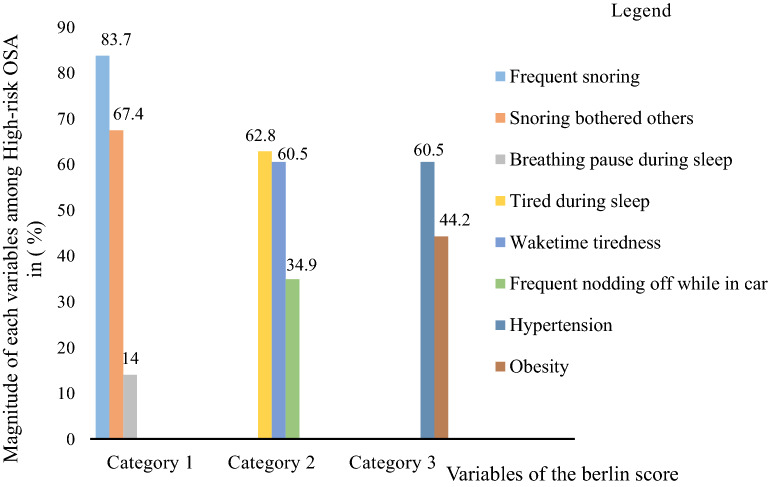
Graphic presentation of variables in berlin score among high-risk OSA T2DM patients (n = 43)

From high-risk OSA T2DM patients 32 (74.4%), 28 (65.1%), and 33 (76.7%) were positive for categories 1, 2, and 3, respectively as compared to low-risk OSA T2DM patients 5 (8.5%), 12 (20.3%), and 16 (27.1%) with p < 0.05. Among high-risk OSA T2DM patients the majority, 36(83.7%) had frequent snoring and 29(80.6%) reported their snoring bothered their bed partners. About 26(60.5%) high-risk OSA T2DM patients had hypertension, and 19(44.2%) were obese. Regarding the tiredness of high-risk OSA T2DM patients, about 27(62.8%), and 26(60.5%) felt tired after sleep and during wake time, respectively (Fig. 2).

### Factors associated with high-risk obstructive sleep apnea

Among all participants, T2DM patients were significantly and independently associated with high-risk OSA (AOR = 3.88, p = 0.004) after controlling age, neck grasp as well as neck and waist circumference which had a p-value < 0.25 in bivariate analysis.

Regarding to factors associated to high-risk OSA among T2DM patients, seven variables; Age (p < 0.001), neck circumference (p = 0.001), neck grasp (p < 0.001), waist circumference (p < 0.001), blood glucose control level (p = 0.057), T2DM medications (p = 0.228) and T2DM-related complications (p < 0.001) had p-value < 0.25 on binary logistic regression analysis, hence included in multiple logistic regression analysis.

In multiple logistic regression analysis; age (AOR = 1.13; 95% CI = 1.04, 1.22; p = 0.002), neck grasp (AOR = 6.48; 95% CI = 1.56, 26.96; p = 0.010), waist circumference (AOR = 4.44; 95% CI = 1.12, 17.61; p = 0.034) and the presence of T2DM-related complications (AOR = 4.18; 95% CI = 1.13, 15.43; p = 0.032) had independent and significant association with high-risk OSA among T2DM patients (Table 5).

**Table 5:** Factors associated with high-risk OSA among T2DM patients in WUSH, southern Ethiopia, 2021

Variable	Category	OSA risk (n, %)		Bivariate analysis	p-value	Multivariate	p-value
		High-risk	Low-risk	COR (CI)		AOR (CI)	
Sex	Male	26 (44.8)	32 (55.2)	1.29 (0.58, 2.87) 1	0.531	_______	_____
	Female	17 (38.6)	27 (61.4)				
Age (years), mean (± SD)		64.9 (9.9)	51.3(10.2)	1.15 (1.09, 1.23)	< 0.001*	1.13 (1.04, 1.22)	0.002**
Marital status	Married	34 (42.5)	46 (57.5)	1.07 (0.41, 2.78) 1	0.894	________	_____
	Others ^a^	9 (40.9)	13 (59.1)				
Occupation	Merchants	20 (41.7)	28 (58.3)	1.55 (0.50, 4.77)	0.633	________	_____
	Housewife	10 (52.6)	9 (47.4)	2.41 (0.64, 9.03)			
	Others ^b^	7 (43.8)	9 (56.2)	1.68 (0.42, 6.72)			
	G/ E	6 (31.6)	13 (68.4)	1			
Educational status	Illiterate	8 (40.0)	12 (60)	1.00 (0.33, 2.99)	0.870	________	_____
	Primary	19 (45.2)	23 (54.8)	1.24 (0.52, 2.98)			
	2^ry^ and above	16 (40.0)	24 (60.0)	1			
Resident	Urban	37 (40.7)	54 (59.3)	0.57 (0.16, 2.01)	0.383	_______	_____
	Rural	6 (54.5)	5 (45.5)				
NC (cm)	High	22 (64.7)	12 (35.3)	4.10 (1.72, 9.81)	0.001*	1.71 (0.27, 10.78)	0.567
	Normal	21 (30.9)	47 (69.1)	1		1	
Neck grasp	Positive	30 (85.7)	5 (14.3)	24.9 (8.1, 76.7) 1	< 0001*	6.48 (1.56, 26.96) 1	0.010**
	Negative	13 (19.4)	54 (80.6)				
WC (cm)	C/ obesity	31 (68.9)	14 (31.1)	8.3 (3.39, 20.35)	< 0.001*	4.44 (1.12, 17.61)	0.034**
	Normal	12 (21.1)	45 (78.9)	1		1	
BGL	Poor control	37 (47.4)	41 (52.6)	2.71 (0.97, 7.55) 1	0.057*	1.39 (0.16, 10.4) 1	0.750
	Good control	6 (25.0)	18 (75.0)				
T2DM duration	> 5 years	23 (46.9)	26 (53.1)	1.46 (0.7, 3.2) 1	0.348	______	____
	≤ 5 years	20 (37.7)	33 (62.3)				
Medication	Insulin & oral	8 (57.1)	6 (42.9)	2.02 (0.65, 6.32) 1	0.228	0.52 (0.08. 3.43)	0.495
	Oral alone	35 (39.8)	53 (60.2)				
Complication	Yes	21 (72.4)	8 (27.6)	6.08 (2.34, 15.82) 1	< 0.001*	4.18 (1.13, 15.43) 1	0.032**
	No	22 (30.1)	51 (69.9)				
Alcohol intake history	Former	7 (43.8)	9 (56.2)	0.87 (0.28, 2.66)	0.401	_______	____
	Current	10 (32.3)	21 (67.7)	0.53 (0.21, 1.33)			
	Never	26 (47.3)	29 (52.7)	1			
Chewing chat	Former	6 (60.0)	4 (40.0)	2.42(0.62, 9.41)	0.404	_______	____
	Current	11 (45.8)	13 (54.2)	1.37 (0.53, 3.5) 1			
	Never	26 (54.2)	42 (45.8)				
Sleeping pills/user	Former	9 (45.0)	11 (55.0)	1.21 (0.44, 3.35)	0.897	_______	____
	Current	9 (45.0)	11 (55.0)	1.21 (0.44, 3.35) 1			
	Never	25 (40.3)	37 (59.7)				

*AOR* adjusted odds ratio, *COR* crude odds ratio, *CI* 95% confidence interval, *OSA* obstructive sleep apnea, *NC* neck circumference, *WC* waist circumference, *BGL* blood glucose level, *T2DM* type 2 diabetes mellitus, *G/E* government employee, *C/ obesity* central obesity, *2*^*ry*^ secondary

^*^association in bivariate analysis, ** significant association on multivariate analysis

^a^(widowed, single and divorced), ^b^(farmers, unemployed), 1(reference)

## Discussion

This is a comparative study in Ethiopia that was conducted to assess OSA risk among T2DM patients by utilizing the Berlin questionnaire. The study disclosed that the magnitude of high-risk OSA among T2DM patients was higher in comparison to age-sex-matched non-diabetic respondents. The study also identified factors: age, neck grasp, waist circumference, and the presence of T2DM-related complications were independent predictors of high-risk OSA among T2DM patients. Although neck circumference was a common independent predictor of high-risk OSA in prior studies [37–40], this study couldn’t find an independent association. The reason could be the gender and size normative neck gap measure might predict better and veneer the predicting ability of neck circumference, which are two parameters that measure the neck mass independently, to predict high-risk OSA.

The prevalence of high-risk OSA among T2DM patients was 42.2%. This result was in line with the studies conducted in Ethiopia (45.5%) [40], Kenya (44.4%) [41], Saudi Arabia (44.3%) [39], and Jordan (48.5%) [38]. This resemblance could be due to similarity in the population studied as well as study tools and procedures: Kenya, Saudi Arabia, and Jordan used the Berlin questionnaire to diagnose high-risk OSA which was similar to the current study. Although the prior study conducted in Ethiopia was different in the study design and tools, the similarity of the population studied might shield the difference. However, the prevalence of high-risk OSA in this study was higher than in studies conducted in Nigeria (27%) [42] and Korea (23%) [37]. This variability might be due to the difference in the population studied, study design as well as variability in study tools and procedures. On the other hand, the prevalence of high-risk OSA among T2DM patients in this study was lower than the study done in Egypt (60%) [43], India (79%) [32], Thailand (75.6%) [29], China (60%) [44], and America (86%) [45]. The possible reasons for the lower prevalence in this study might be because of the different populations studied, the socio-economic status of the study participants, and the tools used to evaluate OSA. For instance, all the study population of India, and America were obese/overweight, which is the common pathway that links OSA and T2DM [46]. Egypt, Thailand, and China used polysomnography for the diagnosis of OSA on hospitalized T2DM patients, in which inpatient cases might be more severe than outpatients who were on regular follow-up.

The study found that there was a significant difference in the prevalence of high-risk OSA among T2DM patients and non-diabetic participants (42.2% vs 13.7%). T2DM patients were nearly four times more likely to have high-risk OSA when compared to the non-diabetic population after controlling for age, neck grasp as well as neck and waist circumference. This significant difference is supported by the comparative study conducted in Kenya (44.4% vs 8%) [41] and India (79% vs 60%) [32]. This significant difference might be because of the bidirectional relationship between T2DM and OSA. T2DM might cause OSA, T2DM might increase predisposition to or accelerate the progression of OSA, possibly through the development of peripheral neuropathy, anatomic alterations, and disturbance in the upper airway neuromuscular control system. Diabetes-associated neuromuscular dysfunction of the upper airway dilator muscle might impair the control of ventilation or impair the control of pharyngeal muscles promoting OSA [46, 47]. Conversely, OSA, through the effects of intermittent hypoxia and sleep fragmentation, could contribute independently to the development of insulin resistance, glucose intolerance by the alteration of the neuroendocrine system mainly via activation of the sympathetic nervous system, hypothalamic-pituitary axis alterations, and changes in the inflammatory pathways leading to T2DM [48, 49].

According to this present study, age was significantly associated with high-risk OSA in which the occurrence of high-risk OSA increased by 13% as age increased by one year. This result was supported by previous studies conducted in India [32], Jordan [38], China [44]. The possible justification might be a generalized age-related decrease in the size of the upper airway lumen. Structural changes to the dimensions of the upper airway include a lengthening of the pharyngeal airway and the descent of the hyoid bone leading to an increase in pharyngeal resistance. Old age is also accompanied by muscular and neurological loss of muscle tone of the upper airway, increased comorbidities as well an increased proportion of fat body mass with age might also account for an increased risk of OSA and enhanced collapsibility [47, 50, 51].

In this study, waist circumference was a significant predictor of high-risk OSA among T2DM patients in which patients with central obesity were more than 4 times the chance to have high-risk OSA as compared to normal waist circumference. This is consistent with studies conducted in Kenya [41], Saudi Arabia [39], Korea [37], and America [45]. The rationale could be fat deposits around the thoracic cage reduce chest compliance and functional residual capacity. Central obesity can also increase pharyngeal collapsibility through mechanical effects on pharyngeal soft tissues and lung volume as well as through the central nervous system–acting signaling proteins (adipokines) that might affect airway neuromuscular control [47, 52].

The current study also identified that neck grasp had an independent association with high-risk OSA among T2DM patients in which the occurrence of high-risk OSA increased over six times in T2DM patients with positive neck grasp (neck gap measured ≥ 1 cm) as compared to negative neck grasp (gap measure below 1 cm). This is in agreement with the prior study reported in America [26]. The reason could be fat deposit around the neck might narrow the upper airway with an increase in soft tissue size (soft palate and tongue), decreased cross-sectional area, and increased compliance of the upper airway which leads to collapsibility of retropalatal and retroglossal airway all contribute to OSA [47, 53].

Finally, this study justified that the presence of T2DM-related complications had a significant association with high-risk OSA. T2DM patients with T2DM-related complications were about four times more likely to have high-risk OSA as compared with patients without complications. This is supported by studies conducted in Egypt [43] and Thailand [29], veteran [54], and china [55]. The possible mechanism could be the metabolic factors mediated by hyperglycemia leading to abnormal nerve energy transport, impaired axonal transport, increased activity of the sorbitol pathway, non-enzymatic nerve protein glucosylation, and abnormal myoinositol metabolism, resulting in neuromuscular dysfunction which might lead to OSA [22].

Conversely, OSA may also worsen certain typical microvascular complications, and be involved in the development of microvascular complications. This close linkage between OSA and microvascular complications in diabetes worsens mainly by intermittent hypoxemia secondary to OSA [54–56]. OSA was found to be associated with increased inflammation, oxidative stress due to repeated hypoxemia-reoxygenation episodes and alterations in nitric oxide synthase activity, increased advanced glycation end product production, increased VEGF, and changes in protein kinase C signaling which plays an important role in the cell response to hypoxia. The final result of this process, aggravated by the effect of hyperglycemia and hypertension, is resulting in the development of endothelial dysfunction and microvascular complications (neuropathy, nephropathy, and retinopathy) [43, 56].

In addition, Intermittent hypoxia and sleep fragmentation caused by OSA are known to activate the sympathetic nervous system and the renin–angiotensin-aldosterone system, increase cytokine levels, and contribute to oxidative stress. The increased generation of free radicals gives rise to several harmful processes, such as endothelial dysfunction, inflammation, platelet aggregation, atherosclerosis, and fibrosis, which may increase the risk of renal damage in patients with OSA. a risk factor for the progression of chronic nephropathy to end-stage renal disease. sleep apnea episodes increase intracranial pressure, which subsequently reduces retinal blood flow and thereby induces retinal injury, leading to diabetic retinopathy [54, 56].

## Limitation of the study

First, the research had been measured to screen high-risk OSA, not OSA due to the lack of PSG machines to diagnose OSA. Second, glycemic control was measured with FBS level rather than the glycated hemoglobin due to budget constraints. Thirdly, due to the nature of the study design, cause-effect relationships of the variables were not assessed. Despite these limitations, the study clearly shows the magnitude of high-risk OSA, significant differences, and its predictors among respondents which have not been investigated in Ethiopia so far.

## Conclusions

This study concluded that the prevalence of high-risk OSA among T2DM patients was higher with a significant difference between T2DM and non-diabetic participants, which was not detected earlier during their routine visits to the hospital. Age, neck grasp, waist circumference, and T2DM-related complications were significantly and independently associated with high-risk OSA among T2DM patients. Therefore, the research outcomes suggest that T2DM patients need to be screened for OSA by health care providers in the early course of their follow-up and treatment to reduce the burden of high-risk OSA and related complications. Physicians should give special attention to T2DM patients with old age, positive neck grasp, central obesity and T2DM-related complications for early notice of high-risk OSA to take preventive care and to minimize further complications.

## Data Availability

The datasets used and/or analyzed during the current study are available from the corresponding author upon reasonable request.

## Abbreviations

AHI: Apnea-hypopnea index
AOR: Adjusted odds ratio
BGL: Blood glucose level
BMI: Body mass index
BP: Blood pressure
CI: Confidence interval
COR: Crude odds ratio
CPAP: Continuous positive airway pressure
DM: Diabetes mellitus
ESAP: Easy sleep apnea predictor
IH: Intermittent hypoxia
NC: Neck circumference
OSA: Obstructive sleep apnea
OPD: Outpatient department
PSG: Polysomnography
SAHS: Sleep apnea-hypopnea syndrome
SD: Standard deviation
SDB: Sleep disorder breathing
SPSS: Statistical Package for social science
T2DM: Type 2 diabetes mellitus
USA: United States of America
WC: Waist circumference
WHO: World Health Organization
WUSH: Wolkite University Specialized Hospital
